# Soil Bacteria to Regulate *Phoebe bournei* Seedling Growth and Sustainable Soil Utilization under NPK Fertilization

**DOI:** 10.3390/plants10091868

**Published:** 2021-09-09

**Authors:** Zhi-Jian Yang, Xiao-Hui Wu, Lan-Ming Huang, Wei-Wei Xie, Yu Chen, Yousry A. El-Kassaby, Jin-Ling Feng

**Affiliations:** 1 College of Forestry, Fujian Agriculture and Forestry University, Fuzhou 350002, China; yangzhijian@fafu.edu.cn (Z.-J.Y.); 3170422017@fafu.edu.cn (X.-H.W.); 3190422012@fafu.edu.cn (L.-M.H.); 3200422020@fafu.edu.cn (Y.C.); 2 Fujian Academy of Forestry Sciences, Fuzhou 350002, China; Weiweixie2020@163.com; 3 Department of Forest and Conservation Sciences, Faculty of Forestry, University of British Columbia, 2424 Main Mall, Vancouver, BC V6T 1Z4, Canada

**Keywords:** soil bacterial, N, P, and K fertilization, seedling cultivation, coupling relationship, marker species

## Abstract

Soil bacteria play a key role in the plant–soil system and can regulate the growth of *Phoebe bournei* seedlings under fertilization. However, there are few reports on how soil bacteria respond to fertilization and regulate seedling growth. This study adopted the “3414” field fertilization experiment, combined with soil microbial sequencing, nutrient contents, and biomass measurement, to explore the changes of soil chemical properties and bacterial structure under different NPK fertilization conditions and to establish the coupling relationship between soil bacteria, soil nutrients, and plant growth. The results showed that NPK fertilization decreased soil pH; increased soil N, P, and K content; reduced bacterial diversity and abundance; promoted the growth of dominant bacterial species; and enhanced *Phoebe bournei* seedlings’ soil N, P, and K elements. NPK fertilization promoted Proteobacteria growth, especially of three genera (*Methylobacterium*, *Sphingobium*, and *Acinetobacter*) and Actinobacteria, while it decreased Acidobacteria and Chloroflexi. By reducing the ratio of N to K and increasing P, NPK fertilization can slow soil acidification, promote bacterial reproduction, maintain *P. bournei* seedlings’ soil ecological stability, and balance the seedlings’ growth and sustainable soil utilization. *AD3*, *Pseudomonas*, and *Rhodanobacter* can be used as the marker species for N, P, and K fertilization, respectively, while *Methylobacterium*, *Brevundimonas*, *Acinetobacter*, and *Sphingobium* can be used as indicator species for soil pH and soil N, P, and K content changes, respectively. These results provided a theoretical basis and technical guidance for the effective fertilization and cultivation of robust *P. bournei* seedlings.

## 1. Introduction

Soil microorganisms, the main driving force of soil material circulation, have a direct relationship with soil fertility and play an essential role in determining soil properties and affecting plant growth [1,2,3]. Bacteria account for about 70~90% of the soil microbial population [4,5] and participate in a variety of soil physical and chemical processes, such as decomposition of soil organic matter, formation of humus, and transformation and circulation of nutrients. Soil bacteria not only perform soil material transformation but also store plant nutrient elements and can be used as the measurement index of soil quality [6,7,8,9]. At the same time, bacteria are sensitive to environmental changes and can also be used as an early indicator of soil changes [10]. Thus, studying soil bacteria is particularly important.

Soil bacteria play an important role in the material circulation and energy flow of the plant–soil system [11]. Changes in the soil environment are expected to lead to a change in bacterial adaptability as well as an effect on its community structure [12]. Fertilization affects the transformation and storage of the available nutrients in soil, leading to changes in the physical and chemical properties of soil, and strongly affects the diversity and abundance of soil bacteria, which, in turn, may influence plant growth and quality [13,14,15]. The addition of NPK fertilizer is expected to influence bacterial community structure, and its impact is not conclusive and somewhat controversial. Research results have indicated that the combined application of NPK fertilizer has no significant effect on soil bacterial diversity [16], while others have argued that it could have a diversity reduction effect [17]. However, inorganic fertilizer can change nutrient-rich and nutrient-poor bacteria in soil, which their relative abundance can be used as a microbial index to indicate and predict soil nutrient content [18,19]. Studying the influence of fertilization on changing soil bacterial community diversity and abundance is of importance in regulating soil microbial community structure, improving plant productivity, and promoting the sustainable utilization of soil.

In addition to soil factors, soil bacteria are also affected by plant types [20]. Soil microorganisms and plants act in a mutualistic manner so that different plants are associated with different enrichment microorganisms [21]. *Phoebe bournei* (Hemsl.) Yang is a rare, endemic, and endangered tree species in China with high economic and ecological value [22]. Bacteria have been reported to be the main soil microorganism component of *P. bournei*, accounting for 98.2~99.8% [23]. *P. bournei* seedling growth (height and ground diameter) can be regulated by the application of bacterial and NPK fertilizers [24,25]; however, few reports on the effects of fertilization on soil bacterial community structure, and how bacteria regulate seedling growth are available.

High-throughput sequencing has facilitated studying bacteria, specifically those species that are difficult to culture, and allowed the generation of information on soil bacterial community structure and growth [26]. Furthermore, the “3414” balanced fertilization protocol, with its advantages of empirical, forest nutrition diagnosis, and formula fertilization methods, offers strong practicability and operability and is widely implemented in fertilization experiments [27]. Here, we combine the use of soil microbial sequencing and the “3414” fertilization protocol to assess nutrient content and biomass in the soil and *P. bournei* seedlings, as well as soil bacterial structure changes under different fertilization conditions. Furthermore, we attempted to establish the coupling relationship between soil bacteria, soil nutrients, and plant growth to identify the key bacteria driving soil nutrient cycling and seedling growth. We anticipate that this work will contribute to our understanding of *P. bournei* seedlings’ living environmental mechanisms, the soil bacteria regulating seedling growth, and the sustainable utilization of soil under N, P, and K fertilization and will provide guidance for the species’ robust cultivation and rational fertilization.

## 2. Results

### 2.1. Effect of NPK Fertilization on Phoebe bournei Seedlings’ Soil Chemical Characteristics

Different NPK fertilization treatments significantly affected the soil pH and N, P, and K contents of *P. bournei* seedlings (Table 1). With the exception of T2, soil N contents of all treatments were higher than the control (T1), with T11 and T2 representing the highest and lowest values, respectively. Soil P contents were lower than the control (T1); however, T3, T6, T8, and T9 produced higher values, with T8 and T4 representing the highest and lowest values, respectively. Soil K contents of all treatments were higher than the control (T1), with T7 and T13 representing the highest and lowest values, respectively. With the exception of T2, soil pH of all treatments was lower than the control (T1). Generally, as determined by the multiple range tests, the three-factor (N, P, and K) fertilizer had the greatest influence on soil pH and N, P, and K contents, followed by two factors and a single factor. In the seven NPK fertilization combinations, N fertilizer had the least effect on soil K content, and, similarly, the P fertilizer had a minimal effect on soil N and P content and pH value.

### 2.2. Analysis of Microbial Species Composition of Phoebe bournei Seedlings’ Soil at the Phylum and Genus Levels

**Figure 1:**
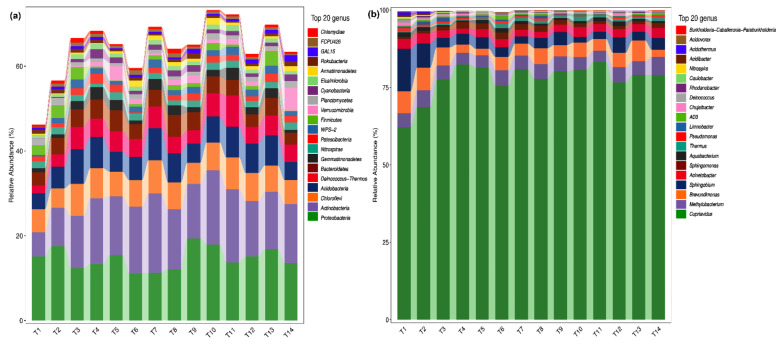
Phylum and genus horizontal species composition in *Phoebe bournei* seedlings’ soil bacteria under different fertilization treatments (T1–T14). (**a**) Genus horizontal; (**b**) phylum horizontal.

The composition and abundance of soil microorganisms under different fertilization treatments were obtained at the taxonomic level of phyla and genera by QIIME software. The top 20 species of relative abundance at the genus level are illustrated in Figure 1a. Among them, 15 genera were with >1% relative abundance, accounting for 61.0% of total genera. These include: *Cupriavidus* (14.6%), *Methylobacterium* (13.7%), *Brevundimonas* (6.3%), *Sphingobium* (6.1%), *Acinetobacter* (4.4%), *Sphingomonas* (3.9%), *Aquabacterium* (1.8%), *Thermus* (1.5%), *Pseudomonas* (1.4%), *Limnobacter* (1.4%), *AD3* (1.4%), *Chujaibacter* (1.3%), *Deinococcus* (1.1%), *Rhodanobacter* (1.1%), and *Caulobacter* (1.0%), indicating that *Cupriavidus*, *Methylobacterium*, *Brevundimonas*, *Sphingobium*, *Acinetobacter*, and *Sphingomonas* were the dominant bacteria genera in *P. bournei* seedlings’ soil. The sum of relative abundance of dominant genera under each fertilization treatment was greater than the control (T1), indicating that NPK fertilization promoted dominant genera bacteria growth. Minimum abundance of *Methylobacterium*, *Sphingobium*, and *Acinetobacter* was observed in the control (T1), indicating that NPK fertilization increased their growth. Maximum abundance of *Cupriavidus*, *Brevundimonas*, and *Sphingomonas* was observed in T9, T7, and T8, while the minimum was observed in T6, T2, and T14, indicating that only an appropriate NPK fertilization ratio could improve their growth. The best *Brevundimonas* and *Sphingomonas* growth was observed under high-P and no-K fertilizer levels, while the worst *Brevundimonas* growth was observed under no-N fertilizer.

At the phylum level, the top 20 species of relative abundance included 7 phyla with >1% relative abundance, accounting for 95.8% of the total. These include: Proteobacteria (77.5%), Actinobacteria (4.6%), Chloroflexi (4.5%), Acidobacteria (4.3%), Deinococcus-Thermus (2.6%), Bacteroidetes (1.3%), and Gemmatimonadetes (1.0%) (Figure 1b). This indicates that Proteobacteria, Actinobacteria, Chloroflexi, and Acidobacteria are the dominant phyla bacteria in *P. bournei* seedlings’ soil. The total abundance of dominant bacteria phyla in each treatment was greater than the control (T1), indicating that NPK fertilization promoted the dominant bacteria phyla growth. Among the 14 fertilization treatments, the maximum abundance of Proteobacteria, Actinobacteria, Chloroflexi, and Acidobacteria was observed in T11, T14, T2, and T1 (control), respectively, and the minimum abundance was observed in T1, T11, T5, and T10, respectively, indicating that NPK fertilization increased Proteobacteria and inhibited Acidobacteria growth and that only an appropriate NPK fertilization ratio can improve Actinobacteria and Chloroflexi growth. The best Chloroflexi growth was observed under no-N fertilizer, while the worst Actinobacteria growth was observed under a high-N level.

### 2.3. Effect of NPK Fertilization on Phoebe bournei Seedlings’ Soil Bacterial Diversity

Different NPK fertilization conditions significantly affected soil microbial Chao1, Simpson, and Shannon indices (Table 2). Chao1, Simpson, and Shannon indices of the control (T1) were higher than all fertilization treatments, indicating that fertilization reduced *Phoebe bournei* seedlings’ soil microbial diversity, with T10 showing the lowest values for the three indices, indicating that soil bacteria diversity was inhibited by a high-K fertilizer level. The three (Chao1, Simpson, and Shannon) diversity indices for the three-factor fertilizer (N, P, and K) were the highest, followed by the two-factor and single-factor fertilizers, with P having the least effect on soil diversity indices.

### 2.4. Differential Groups of Phoebe bournei Seedlings’ Soil Bacteria under NPK Fertilization

**Figure 2:**
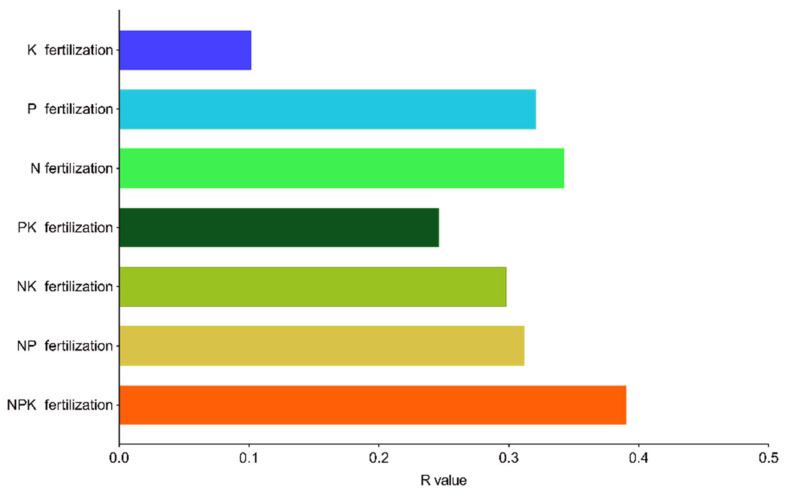
Anosim significance test of community structure difference between *Phoebe bournei* seedlings’ different fertilization groups. R-value ranges between −1 and 1 (values >0 indicate a significant difference between groups).

The Anosim significance test showed that N, P, and K three-, two-, and single-factor fertilization treatments had significantly different impacts on soil bacterial community structure, with the following order: NPK, N, P, NP, NK, PK, and K fertilization (Figure 2).

#### 2.4.1. Differential Soil Bacteria Groups under Three-Factor NPK Fertilization

**Figure 3:**
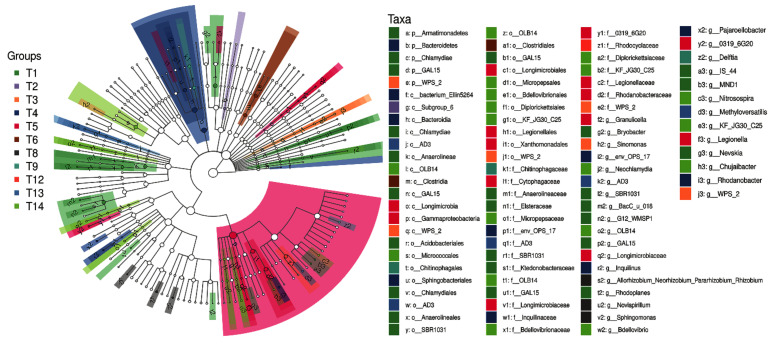
Analysis of soil bacterial differences of *Phoebe bournei* seedlings under NPK fertilization treatments.

We used Lefse1.0 software to compare the species composition differences among the 14 NPK treatments to identify the marker species. The results showed that at the taxonomic phylum level, the marker species were Bacterodetes, Armatimonadetes, Chlamydiae, WPS-2, and Gal15. At the genus level, the top 10 marker species among treatments were *Granulicella*, *Env-OP-17*, *Sinomonas*, *OLB14*, *SBR1031*, *Pajarollobacter*, *F-0319-6G20*, *Sphingononas*, *Allorhizobium-Neorhizobium-Pararhizobium-Rhizobium*, *Rhodanobacter*, and *G-AD3* (Figure 3).

#### 2.4.2. Differential Soil Bacteria Groups under Two-Factor NPK Fertilization

**Figure 4:**
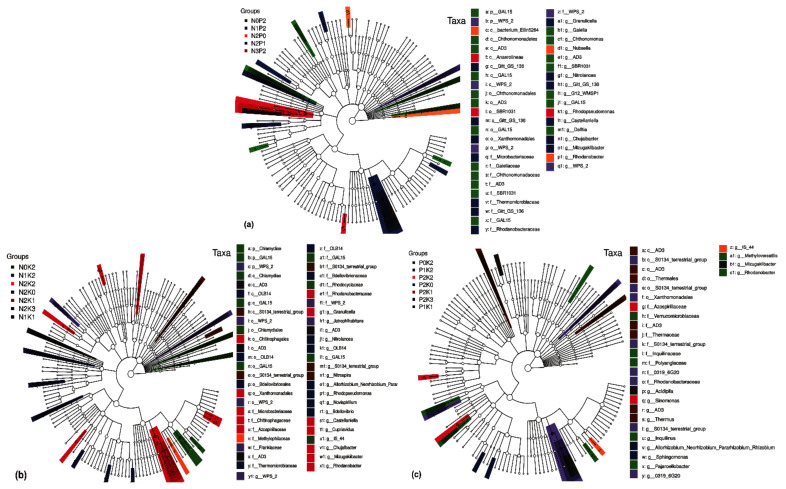
Analysis of soil bacterial differences of *Phoebe bournei* seedlings under two-factor NPK fertilization treatments. (**a**) NP fertilizer application rate changes; (**b**) NK fertilizer application rate changes; (**c**) PK fertilizer application rate changes.

Among NP fertilization treatments and at the phylum and genus levels, we identified the following marker species: WPS-2 and Gal15 and *AD3*, SBR1031, *Nitrolancea*, *Gitt_GS_136*, *G12_WMSP1*, *GAL15*, *Rhodopseudomonas*, *Castellaniella*, *Delftia*, *Chujaibacter*, *Mizugakibacter*, and *WPS-2*, respectively (Figure 4a). Similarly, among NK fertilization treatments, we identified Chlamydiae, WPS-2, and GAL15 species at the phylum level and *Granulicella*, *Jatrophihabitans*, *AD3*, *Nitrolancea*, *OLB14*, *GAL15*, *S0134_terrestrial_group*, *Nitrospira*, *Novispirillum*, *Bdellovibrio*, *Castellaniella*, *Cupriavidus*, *Rhodanobacter*, and *Mizugakibacter*, *Allorhizobium_Neorhizobium_Pararhizot_Rhodopseudomonas* at the genus level (Figure 4b). For PK fertilization treatments, we identified *Acidipila*, *Sinomonas*, *Thermus*, *S0134_terrestrial_group*, *Inquilinus*, *0319_6G20*, *IS_44*, *AD3*, *Allorhizobium_Neorhizobium_Pararhizobium_Rhizobium*, *Sphingomonas*, *Pajaroellobacter*, *Methyloversatilis*, *Mizugakibacter*, and *Rhodanobacter* as marker species at the genus level (Figure 4c).

#### 2.4.3. Differential Soil Bacteria Groups under Single-Factor NPK Fertilization

**Figure 5:**
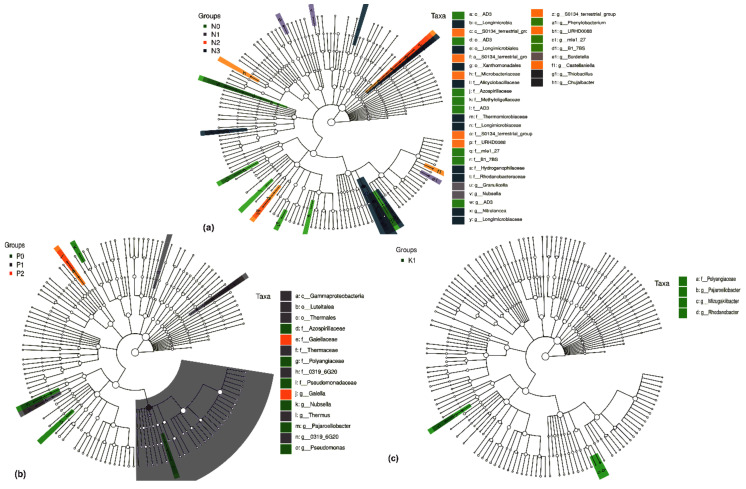
Analysis of soil bacterial differences of *Phoebe bournei* seedlings under single-factor NPK fertilization treatments. (**a**) N fertilizer application rate changes; (**b**) P fertilizer application rate changes; (**c**) K fertilizer application rate changes.

Among N fertilization treatments, we identified Granulicella, Nubsella, AD3, Nitrolancea, Longimicrobiaceae, S0134_terrestrial_group, Phenylobacterium, URHD0088, mle1_27, B1_7BS, Bordetella, Castellaniella, Thiobacillus, and Chujaibacter as marker species at the genus level (Figure 5a). Similarly, among P and K fertilization treatments, we identified Gaiella, Nubsella, Thermus, Pajaroellobacter, 0319_6G20, and Pseudomonas (Figure 5b) and Pajaroellobacter, Mizugakibacter, and Rhodanobacter as marker species at the genus level (Figure 5c), respectively.

### 2.5. Network Association Analysis of Phoebe bournei Seedlings’ Soil Microbial Community under NPK Fertilization

Network modularity segmentation of bacterial communities in *Phoebe bournei* seedlings soil was performed under the various fertilization conditions (Table 3). The modularity index was greater than 0.7 for the seven NPK fertilization combinations, indicating that the community had a modular structure. Number of nodes, number of edges, and closeness centrality in bacterial community were the highest under NPK, NP, and NK, followed by N, PK, K, and P, fertilization. Transitivity coefficient and average path length in the bacterial ecosystem under P was the highest, followed by K, PK, the remaining three fertilization conditions (NPK, NP, and NK), and N fertilization, indicating that single-factor fertilization was the lowest in network stability, followed by two-factor fertilization; the highest stability was with three-factor fertilization. Under single-factor fertilization, N was the most stable network, followed by K and P; this could be due to the improved diversification of three-factor fertilization, leading to enhanced interaction and stability among bacteria.

**Figure 6:**
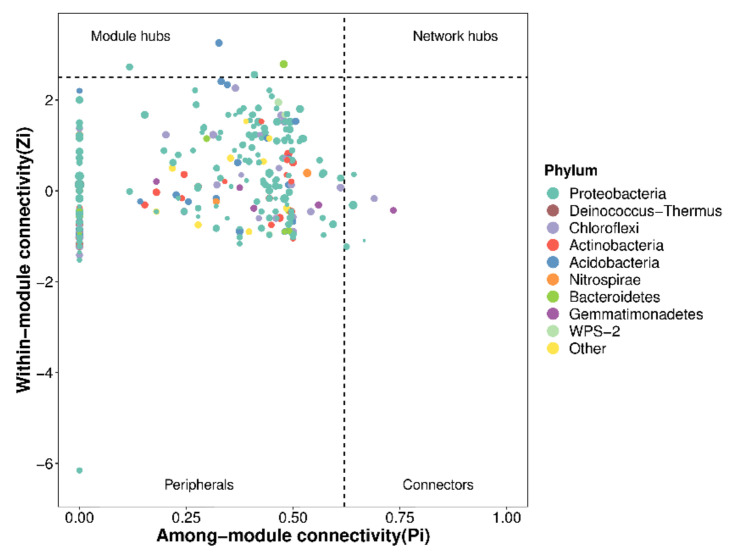
Scatter plots of soil bacterial marker species of *Phoebe bournei* seedlings under NPK fertilization treatments. The node in the network can be divided into four parts using Zi and Pi values, namely, peripherals, connectors, module hubs, and network hubs. Peripherals represent some specialists in microbial networks. Module hubs and connectors represent species that are close to generalists. Network hubs represent super-generalists among the microbial networks.

The network association among microbial members was visualized using the R graph package, and nine key phylum species were found in different NPK fertilization treatments, including Proteobacteria, Deinococcy-Thermus, Chloroflexi, Actinobacteria, Acidobacteria, Nitrospirae, Bacteroidetes, Gematimonadetes, and WPS-2 (Figure 6).

### 2.6. Driving Factors of Phoebe bournei Seedling Growth under NPK Fertilization

**Figure 7:**
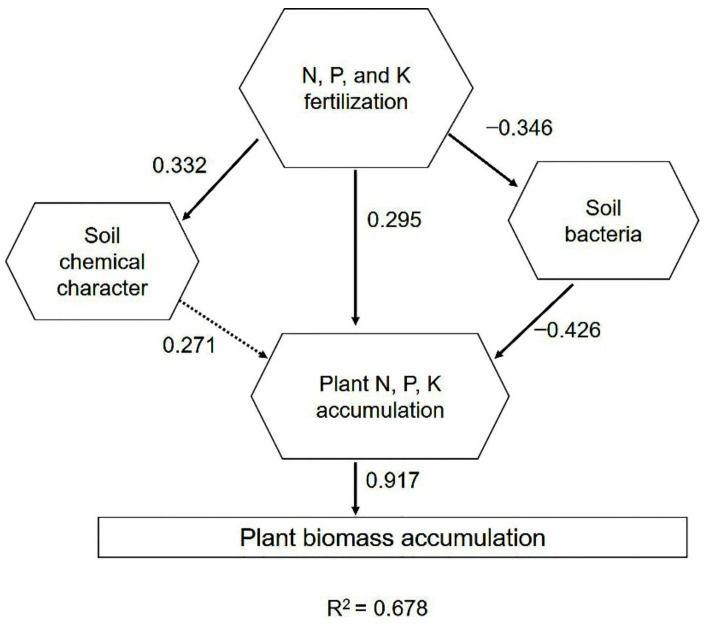
Complex interrelationships of soil bacteria with soil chemical properties, seedlings nutrient and biomass accumulation under NPK fertilization by the partial least squares path mode. Black solid and dashed lines represent significant effects (*p* < 0.05) and non-significant effects (*p* > 0.05), respectively. The data represent the magnitude and direction of the association between the two systems.

PLS-PM results showed that the growth of *Phoebe bournei* seedlings was significantly affected by NPK accumulation, which was driven by three aspects, namely, the significant and positive effect of NPK fertilizers, the positive effect of soil chemical properties, and the negative effect of bacteria growth. According to the absolute value of the correlation coefficient, the factors influencing *P. bournei* seedling growth were soil bacteria, soil chemical character, and NPK fertilization, indicating soil bacteria was the most important factor for *P. bournei* seedling growth (Figure 7). Further correlation analysis of soil dominant phylum and genera bacteria indicated that *Methylobacterium*, *Brevundimonas*, *Sphingobium*, and *Acinetobacter* had significant or highly significant negative correlation with Actinobacteria, Chloroflexi and Acidobacteria and significant or highly significant positive correlation with Proteobacteria, which indicated that *Methylobacterium*, *Brevundimonas*, *Sphingobium*, and *Acinetobacter* were the key species regulating the soil ecology of *Phoebe bournei* seedling soil (Table 4).

Correlation analysis of soil key bacteria and chemical traits showed that pH was negatively correlated with *Methylobacterium*, *Brevundimonas*, *Sphingobium*, and *Acinetobacter* abundance, among which significant correlation with observed for *Methylobacterium* and *Acinetobacter*. Soil NPK contents were positively and significantly correlated with the abundance of four key bacteria (N content with *Brevundimonas* and *Acinetobacter*, P content with *Acinetobacter*, and K content with *Sphingobium*) (Table 5).

## 3. Discussion

### 3.1. NPK Fertilization Regulation of Phoebe bournei Seedlings’ Soil Chemical Characters

Fertilization is the main influencing factor for soil properties, causing changes in soil nutrients and pH [28]. NPK fertilization reduced *P. bournei* seedlings’ soil pH, with the largest change under N and the least under P; these results are consistent with previous observations [29,30]. NPK fertilization can reduce soil acidification by decreasing and increasing the proportion of N and P, respectively. NPK fertilization significantly increased *P. bournei* seedlings’ soil N and K contents, possibly due to promoting N- and K-rich microorganism growth. Soil P content can be significantly increased only when appropriate NPK application is applied, especially by reducing the K ratio. K fertilizer increased soil K content and the amount of soil H^+^ due to the increased competition between the binding points of H^+^ and K^+^ on the plasma membrane, thus enhancing P activation capacity [31,32,33] and, consequently, increasing *P. bournei* seedlings’ P uptake, resulting in reduced soil P content. Hence, soil P content in T8 (N_2_P_2_K_0_), T9 (N_2_P_2_K_1_), and T6 (N_2_P_2_K_2_) was higher than that of T1 (N_0_P_0_K_0_), with T8 representing the highest soil P content among the 14 fertilization treatments. Multiple range analysis showed that the three-factor NPK fertilization had the greatest influence on soil NPK content, followed by double-factor and single-factor fertilization, indicating that NPK could improve the sustainable soil productivity of *P. bournei* seedlings. P appeared to be the key factor in regulating *P. bournei* seedlings’ stem growth [34]. Therefore, reducing the K fertilization proportion in NPK fertilizer combinations can improve the seedlings’ P uptake.

### 3.2. NPK Fertilization Regulation of Phoebe bournei Seedlings’ Soil Bacterial Diversity

Soil bacterial diversity is closely related to soil functions. Protecting soil bacterial diversity can effectively ensure the existence of a balanced and stable soil ecosystem [13,14,15]. Fertilization level, type, duration, and application method can individually and/or in concert change soil physical and chemical properties and, subsequently, affect soil bacteria species and diversity [17,35]. In the present experiment, NPK fertilizer acidized the soil and increased its nutrient content, resulting in the enrichment of some soil bacterial growth along with a reduction in their diversity; these results support previous observations [17,18,19]. Soil bacterial diversity under three-factor fertilization was higher than two- and single-factor fertilization, indicating that three-factor fertilization provided the most balanced nutrients for *P. bournei* seedlings’ soil bacteria and satisfied the growth and metabolism requirements of more bacteria types, a keeping high diversity level. At the same time and as previously reported, P fertilizer had the least effect on soil bacteria diversity [36]. Therefore, reducing the ratio of N and K and increasing P can maintain *P. bournei* seedlings’ soil ecological stability under NPK fertilization.

### 3.3. Soil Bacteria Response to Phoebe bournei Seedlings’ NPK Fertilization

NPK fertilization significantly affected *P. bournei* seedlings’ soil bacterial community composition. The abundance and network correlation analyses of soil bacteria indicated that Proteobacteria, Actinobacteria, Chloroflexi, and Acidobacteria were the dominant and core phylum species, playing an important role in soil material circulation and ecological environment balance [37,38]. NPK fertilization significantly increased soil fertility and the available N and P contents in the soil, resulting in increased Proteobacteria abundance, as affected by the available N, and decreased Acidobacteria abundance, mainly due to poor soil environment [39,40]. Urea, as the N source in our experiment, was conducive to increasing *P. bournei* seedlings’ nitrite absorption and reducing the nitrite content in the soil, thus decreasing Chloroflexi abundance, which oxidizes nitrites into nitrates, increasing autotrophic nitrite bacteria growth (e.g., the best Chloroflexi growth was in T2, which did not include N fertilizer) [41,42]. Actinobacteria are known to be beneficial bacteria that produce agricultural antibiotics and fight plant diseases [43]. Similar to most NPK fertilization results, high N levels resulted in reduced Actinobacteria abundance; however, an appropriate NPK ratio can promote Actinobacteria growth, reaching maximum abundance in T14 (N_2_P_1_K_1_), probably because NPK fertilizer provides material and energy in relatively arid red soil [40,44]. *Cupriavidus*, *Methylobacterium*, *Brevundimonas*, *Sphingobium*, *Acinetobacter*, and *Sphingomonas* were the dominant genera in *Phoebe bournei* seedling soil, and they all belong to Proteobacteria, which indicates that Proteobacteria have strong adaptability to NPK fertilization. *Methylobacterium*, *Sphingobium*, and *Acinetobacter* can fully absorb soil nutrients, providing NPK fertilization as substances and energy to promote their reproduction. However, *Brevundimona* propagated best under the application of high-level P fertilizer (T7, N_2_P_3_K_2_), indicating that it had a high phosphorus-dissolving function [45]. *Sphingomonas* grew best without K fertilizer (T8, N_2_P_2_K_0_), indicating that it had a weak potassium-releasing capacity [46].

Soil bacteria can reflect changes in soil in time, such as soil nutrients and pH value, and can temporarily reflect the quality of soil [18,19,28,47]. With a relative abundance of more than 1%, Bacteroidetes was only used as the marker phyla species for NPK three-factor fertilization, while there was no marker phyla species for two- and single-factor fertilization, indicating that the phyla species could not be used as the marker species for NPK fertilization. Under NPK three-factor fertilization, *Rhodanobacter*, *Sphingononas*, and *AD3* were used as the marker genera species, with a relative abundance of more than 1%. Under two-factor fertilization, *AD3*, *Pseudomonas*, and *Chujaibacter* were used as the marker genera species, with a relative abundance of more than 1% for N and P fertilization; *AD3*, *Pseudomonas*, *Cupriavidus*, and *Rhodanobacter* for N and K fertilization; and *Thermus*, *AD3*, *Sphingomonas*, and *Rhodanobacter* for P and K fertilization. Under single-factor fertilization, *AD3* was the marker bacteria for N, with relative abundance over 1%; *Thermus* and *Pseudomonas* for P fertilizers; and *Rhodanobacter* for K fertilizer. These indicate that the genera species can be used as the marker species for NPK fertilization.

### 3.4. Soil Bacteria Regulate P. bournei Seedling Growth and Soil Development

Soil microorganisms play an important role in soil nutrient cycling and the soil–plant growth system [11]. Plants, soil, and microorganisms are not independent in soil material circulation and energy exchange processes but interact with each other in a synergetic manner. NPK fertilization significantly increased soil N, P, and K contents, directly promoting *P. bournei* seedling growth and indirectly improving soil bacteria growth. On the other hand, NPK fertilization directly and significantly inhibited soil bacteria growth; however, it did promote dominant bacteria growth by enriching nutrient elements, promoting N, P, and K element storage in the soil bank, and providing nutrients for subsequent seedling growth. Hence, soil bacteria was the most important factor for *P. bournei* seedling growth and the sustainable utilization of soil. *Methylobacterium*, *Brevundimonas*, *Sphingobium*, and *Acinetobacter* are the key species for regulating soil fertilization. *Methylobacterium* and *Acinetobacte* had a significant negative correlation with soil pH, *Brevundimonas* a significant positive correlation with soil N content, *Sphingobium* with soil K content, and *Acinetobacter* with soil N and P contents, which indicated that *Methylobacterium* and *Acinetobacter* could be used as indicator species of soil pH, *Brevundimonas* as an indicator of soil N content, *Acinetobacter* as an indicator of soil P content, and *Sphingobium* as an indicator of soil K content. Therefore, improving *Brevundimonas*, *Sphingobobacter*, and *Acinetobacter* abundance can increase N, P, and K contents in the soil bank and sustainably provide nutrients for *P. bournei* seedling growth.

## 4. Materials and Methods

### 4.1. Site Description

The experiment site was at a field nursery of the Fujian Agriculture and Forestry University, China (119°23′ E, 26°09′ N). The annual average temperature is 19.6 °C, the minimum temperature is −2.5, and the maximum temperature is 42.3 °C. Effective accumulated temperature ≥10 °C is 5880 °C, with 326 days >0 °C. Annual precipitation is 1490 mm, and annual average humidity is 77% [48].

### 4.2. Materials

Well-grown, well-developed buds and one-year-old *Phoebe bournei* bareroot seedlings were selected for the experimental population in May 2018. The average height of the seedlings was 20.2 ± 0.9 cm, and the average diameter was 2.3 ± 1.0 mm. Seedlings had total N, P, and K contents of 1.219, 1.555, and 14.022 g·Kg^−1^, respectively, at the start of the experiment [48].

Red laterite soil, vermiculite, and sand were used to mix the soil substrate, the volume ratio of which was 6:2:2. Organic matter was 5.78 g·kg^−1^, pH was 5.30, and total N, P, and K contents were 1.5, 0.035, and 33.91 g·kg^−1^ in the soil mixture, respectively. Plastic-pot-grown seedlings (diameter × height: 25 × 25 cm) had 6.0 kg of dry soil. The fertilizers used were urea (N: 47%), superphosphate (P_2_O_5_: 12%), and potassium chloride (K_2_O: 60%) [48].

### 4.3. Experimental Design

The test followed the “3414” fertilizer experimental design, which is set as three factors of N, P, and K [27,49]. Every factor has 0, 1, 2, 3 fertilization levels, respectively. Level 0 is the control (no fertilizer). Level 2 (medium level) is the common fertilizer rates, which were 0.532, 0.133, and 0.356 g·plant^−1^ for N, P_2_O_5_, and K_2_O, respectively [50,51]. Level 1 (low level) and Level 3 (high level) are 0.5 and 1.5 times Level 2, respectively. The specific rates of fertilizer were calculated in terms of N, P_2_O_5_, and K_2_O (Table 6). The “3414” fertilizer experiment has 14 treatment combinations and can analyze the single-factor, two-factor, and three-factor interaction effects of NPK fertilization. In the single-factor effect treatments, when the “2” level was fixed by P and K fertilizers, the 0, 1, 2, 3 levels of N fertilizer were T2, T3, T6, and T11, respectively. When the “2” level was fixed by N and K fertilizers, the 0, 1, 2, 3 levels of P fertilizer were T4, T5, T6, and T7, respectively. When the “2” level was fixed by N and K fertilizers, the 0, 1, 2, 3 levels of K fertilizer were T8, T9, T6, and T10, respectively. In the two-factor interaction treatments, when K fertilizer was fixed at the “2” level, the N–P interactions had 8 treatments, which were T2, T3, T4, T5, T6, T7, T11, and T12. When the “2” level was fixed by P fertilizer, the N–K interaction had 8 treatments, which were T2, T3, T6, T8, T9, T10, T11, and T13. When the N fertilizer was fixed at the “2” level, the P–K interaction had 8 treatments, which were T4, T5, T6, T7, T8, T9, T10, and T14. The three-factor interaction of N × P × K was the T1–T14 treatments. The experiment had 14 treatments, with each treatment replicated three times, and 1260 seedlings, with 30 seedlings at each treatment replication. Random arrangement (complete randomized design) was used for the fertilizer replications to minimize any environmental effects [48].

### 4.4. Experimental Management

This field experiment began in March 2018 and ended in December 2018. P fertilizer was used as the base fertilizer, and N fertilizer and K fertilizer were applied in different stages under the annual growth characteristics of *P. bournei* seedlings [52]. The applied fertilization regime was April—25% N and 20% K, June—35% N and 25% K, August—25% N and 35% K, and October—15% N and 20% K through a liquid application of fertilizer. Additionally, the concentration of fertilizer liquid was 0.05%. *P. bournei* seedlings need a light intensity equivalent of 75% natural light, and a 2.7 m high shed was equipped in the nursery. Seedlings were watered on a varying schedule determined by the empirical method of topsoil coloration, resulting in watering at ~7 days during March to April, ~15 days during May to July, not watered in August, ~10 days during September to November, and ~15 days in December, to maintain the desired soil water status of ~75% field capacity [48].

### 4.5. Sample Collection and Processing

Nine seedlings per treatment were harvested after growth cessation in December 2018. The intact root systems were dug up and gently shaken to harvest the loose soil on the root surface, which were the soil samples. Soil from 9 plants were mixed in each treatment and divided into two parts, and one part was placed in a 5 mL sterile centrifuge tube and stored in a −80 °C refrigerator for soil bacterial sequencing use. The other part was air-dried and screened for chemical indicators.

### 4.6. Soil and Seedling Measurement Parameters

The sampled seedlings were washed with water and dried in the shade, fixed at 105 ℃ for 15 min, and dried at 75 °C to achieve a stable weight. The samples’ dry biomass was measured with 0.001 g accuracy by electronic scales (AL204, Mettler-Toledo, Melbourne, Australia) [48]. Seedlings were prepared for analytical sampling by grinding with a plant crusher and then processing the material through a 0.5 mm plastic sieve. Total N, P, and K of seedlings were dissolved with the H_2_SO_4_-HClO_4_ method, soil total P and K with the NaOH solution-melting method, and soil total N with concentrated sulfuric acid and mixed catalyst (K_2_SO_4_: CuSO_4_ = 10:1). N, P, and K contents were determined by the Kjeldahl method (ATN-300, Hongji, Shanghai, China) [53], the molybdenum-antimony colorimetric method (UV-2600A, Unicom, Shanghai, China) [54], and the atomic absorption spectrophotometer method (AA7002, Dongxi, Beijing, China) [55], respectively. Each indicator was repeated three times. Plant N (P, K) accumulations (mg·plant^−1^) were obtained by multiplying dry biomass (g·plant^−1^) by N (P, K) content (g·Kg^−1^). Plant biomass and the N, P, and K accumulation of each fertilizer combination are listed in Table 7.

### 4.7. Soil Bacterial Sequencing

Soil samples of 0.5 g were used to extract soil DNA using the Fast DNA^®^ Spin Kit for Soil (MP Biomedicals, Carlsbad, CA, USA). The extracted DNA products were tested by agarose gel electrophoresis, and the qualified DNA samples stored at −80 °C until further use. PCR conditions were 30 cycles at 98 °C pre-denaturation for 1 min, 98 °C denaturation for 10 s, 50 °C annealing for 30 s, 72 °C extending for 60 s, and finally extending 72 °C for another 5 min. PCR amplifications of the V3–V4 highly variable region of the bacterial 16S rRNA gene were performed with primers 338F (5′-ACTCCTACGGGAGCAG-3′) and 806R (5′-GGACTACHVGGGTWTCTAAT-3′). After amplification, the recovered products were quantified by Quanti Fluor TM fluorometer, denatured by NaOH into single chains, connected to sequencing joints, and sequenced on PE251 mode of Hiseq2500; a sequencing library was constructed according to Illumina instructions. The 16S rRNA sequence bases were analyzed using the Quantitative Insights into Microbial Ecology (QIIME) platform. Sequences with quality lower than Q20 and lengths less than 200 bp were removed and matched with samples according to the barcode sequence. Amplified primers and joint sequences were removed by Cutadapt and TrimMomatic software, and the Usearch algorithm was used to cluster the sequences according to 97% similarity and to remove chimeric operational taxonomic units (OTUs). Samples were divided into different OTUs; among each of them, that the sequence with the highest abundance was selected as the representative sequence.

### 4.8. Data Analysis

The annotated species in the Silva (Release132, http://www.arb-silva.de) database were selected (10 September 2020), and cluster comparisons were performed on all valid sequences to get OTUs with a >97% sequence similarity level. In this experiment, on average, each sample obtained 593 OTUs, belonging to 23 phyla, 51 classes, 107 orders, 155 families, and 208 genera. Mothur software was used to analyze the abundance and *Alpha* and *Beta* diversity of OTUs in each treatment soil. *Alpha* diversity was assessed by Chao1, Shannon, and Simpson indices. Chao1, the estimated number of OTUs, is defined as

chao1 = S_obs_ + F1(F1−1)/2(F2 + 1)

where S_obs_ are the number of OTUs observed, and F1 and F2 are the counts of singletons and doubletons, respectively [56,57].

Shannon’s index is defined as

$$\mathrm{Shannon}=-\sum_{i=1}^{s}\left(p_{i}{\;\log}_{2}p_{i}\right)$$

where *s* is the number of OTUs, and *p*_i_ is the proportion of the community represented by OUT_i_ [56,57].

Simpson’s index is defined as

$$\mathrm{Simpson}=1-\sum p_{i}^{2}$$

where *p*_i_ is the proportion of the community represented by OUT_i_ [56,58].

Additionally, *Beta* diversity was analyzed by the Anosim test, which was carried out on soil bacteria of all experimental treatments using R language [57,58]. Lefse1.0 software was used to further compare the composition differences of bacterial species among the different treatments and to find biomarker species [58]. The partial least squares path mode (PLS-PM) was used to analyze the relationships between fertilization, soil microorganisms, soil nutrition, and plant nutrition and growth by R language [59]. The results were drawn by Adobe Photoshop CC. Analysis of variance (ANOVA) was used to determine whether soil chemical characteristics and Alpha diversity indices of soil bacterial were significant in all treatments. Then Duncan’s multiple comparisons (α = 0.05) were used to analyze whether there were significant differences between the treatments by SPSS 22.0 (Chicago, IL, USA). Excel 2016 and SPSS 22.0 (Chicago, IL, USA) were used to conduct the range analyses, which were the differences between the maximum and minimum values at different levels for the single-factor, two-factor, and three-factor effects of N, P, and K fertilizer. The correlation analysis between dominant phyla bacteria and dominant genera bacteria and key bacteria and soil chemical characteristics was performed by SPSS 22.0 (Chicago, IL, USA) for the three-factor interaction effect of N, P, and K fertilizer.

## 5. Conclusions

NPK fertilization reduced *Phoebe bournei* seedlings’ soil pH, with the largest effect under N and the least under P for single-factor fertilization. By reducing the rate of N and increasing P, soil acidification can be controlled with minimal pH reduction. NPK fertilization significantly increased soil’s N and K contents and decreased bacterial diversity. With proper NPK application, by reducing the N and K ratios and increasing P, not only can *P. bournei* seedlings be provided with balanced nutrients, but various bacteria can meet their growth and metabolism needs while keeping ecological stability and maintaining seedling growth and a soil sustainable utilization balance. Proteobacteria, Actinobacteria, Chloroflexi, and Acidobacteria were the dominant and core phylum species, while *Cupriavidus*, *Methylobacterium*, *Brevundimonas*, *Sphingobium*, *Acinetobacter*, and *Sphingomonas* were the dominant genus species in *Phoebe bournei* seedlings soil. NPK fertilization significantly affected soil bacterial community composition, increasing Proteobacteria, *Methylobacterium*, *Sphingobium*, and *Acinetobacter* abundance, decreasing Acidobacteria, and promoting Actinomycetes and Chloroflexi under appropriate NPK ratios, *Brevundimonas* under high P level, and *Sphingomonas* in the absence of K. NPK fertilization significantly increased *P. bournei* seedlings’ soil N, P, K contents and indirectly promoted soil bacteria growth. However, it directly and significantly inhibited soil bacteria growth while promoting dominant bacterial species growth (which enrich nutrient elements) and N, P and K element storage in the soil bank (which provide the nutrients needed for subsequent seedling growth). Soil bacteria was the most important factor for *P. bournei* seedling growth and the sustainable utilization of soil. *Methylobacterium*, *Brevundimonas*, *Sphingobium,* and *Acinetobacter* were the key species in *P. bournei* seedlings’ soil under fertilization, with increasing *Brevundimonas*, *Sphingobium,* and *Acinetobacter* abundance inducing increased N, P, and K contents in the soil bank, which sustainably provided the nutrients needed for seedling growth. *AD3* can be used as the marker species under N fertilizer application, *Pseudomonas* under K fertilizer application, and *Rhodanobacter* under K fertilizer application; *Methylobacterium* and *Acinetobacter* for mentoring soil pH; *Brevundimonas* for soil N content; *Sphingobium* for soil K content; and *Acinetobacter* for soil P content change.

## Figures and Tables

**Table 1:** Effect of NPK fertilization on soil chemical characteristics of *Phoebe bournei* seedlings.

Number	Treatment	N Content (g·Kg^−1^)	P Content (g·Kg^−1^)	K Content (g·Kg^−1^)	pH
T1	N_0_P_0_K_0_	2.86 ± 0.35 i	0.35 ± 0.13 ef	32.91 ± 1.67 g	5.29 ± 0.01 b
T2	N_0_P_2_K_2_	2.45 ± 0.09 j	0.18 ± 0.01 gh	51.91 ± 2.63 cd	5.59 ± 0.07 a
T3	N_1_P_2_K_2_	11.73 ± 0.33 b	0.74 ± 0.01 c	49.92 ± 3.88 de	4.74 ± 0.01 e
T4	N_2_P_0_K_2_	4.68 ± 0.04 h	0.16 ± 0.04 h	42.79 ± 0.24 ef	4.43 ± 0.03 h
T5	N_2_P_1_K_2_	5.17 ± 0.26 g	0.25 ± 0.06 fgh	58.88 ± 4.13 bc	4.53 ± 0.04 g
T6	N_2_P_2_K_2_	6.34 ± 0.19 ef	0.50 ± 0.08 d	39.18 ± 1.45 fg	4.65 ± 0.04 f
T7	N_2_P_3_K_2_	4.83 ± 0.14 h	0.24 ± 0.02 fgh	69.39 ± 5.50 a	4.83 ± 0.07 d
T8	N_2_P_2_K_0_	8.20 ± 0.08 c	0.91 ± 0.13 b	58.19 ± 7.34 bc	4.97 ± 0.02 c
T9	N_2_P_2_K_1_	6.50 ± 0.09 e	0.45 ± 0.04 de	48.48 ± 0.58 de	4.84 ± 0.03 d
T10	N_2_P_2_K_3_	5.29 ± 0.08 g	0.28 ± 0.05 fgh	43.67 ± 9.15 ef	4.15 ± 0.04 j
T11	N_3_P_2_K_2_	13.47 ± 0.26 a	2.33 ± 0.18 a	47.41 ± 3.08 de	4.64 ± 0.01 f
T12	N_1_P_1_K_2_	6.15 ± 0.12 f	0.18 ± 0.02 gh	61.86 ± 4.11 b	4.45 ± 0.04 h
T13	N_1_P_2_K_1_	2.79 ± 0.03 i	0.35 ± 0.00 ef	33.70 ± 2.50 g	4.47 ± 0.01 gh
T14	N_2_P_1_K_1_	7.42 ± 0.10 d	0.31 ± 0.04 fg	34.71 ± 3.04 g	4.27 ± 0.02 i
R_N_	–	11.02	2.15	12.72	0.95
R_P_	–	1.66	0.34	30.21	0.40
R_K_	–	2.90	0.62	19.01	0.82
R_NP_	–	11.02	2.17	30.21	1.16
R_NK_	–	11.02	2.15	25.50	1.44
R_PK_	–	3.52	0.75	34.68	0.82
R_NPK_	–	11.02	2.17	36.69	1.44

Means ± SD with different letters indicate a significant difference among the 14 treatments, as determined by Duncan’s multiple range test (*p* < 0.05). R means the difference between the maximum and minimum values at different levels for single-factor, two-factor, and three-factor effects of N, P, and K fertilizer. R_N_ is the difference at 4 N levels (T2, T3, T6, and T11), R_P_ at 4 P levels (T4, T5, T6, and T7), R_K_ at 4 K levels (T8, T9, T6, and T10), R_NP_ at 8 NP levels (T2, T3, T4, T5, T6, T7, T11, and T12), R_NK_ at 8 NK levels (T2, T3, T6, T8, T9, T10, T11, and T13), R_PK_ at 8 PK levels (T4, T5, T6, T7, T8, T9, T10, and T14), and R_NPK_ at 14 NPK levels (T1–T14).

**Table 2:** Effect of different fertilizer treatments on soil alpha diversity indices of soil bacterial in *Phoebe bournei* seedlings.

Number	Treatment	Diversity Index		
		Chao1	Simpson	Shannon
T1	N_0_P_0_K_0_	1929.96 ± 84.55 a	0.97 ± 0.01 a	8.37 ± 0.44 a
T2	N_0_P_2_K_2_	1577.62 ± 152.83 ab	0.96 ± 0.02 ab	7.60 ± 0.57 ab
T3	N_1_P_2_K_2_	1389.66 ± 166.04 ab	0.96 ± 0.02 ab	7.10 ± 0.67 ab
T4	N_2_P_0_K_2_	1102.84 ± 142.98 b	0.95 ± 0.01 ab	6.69 ± 0.43 b
T5	N_2_P_1_K_2_	1048.23 ± 87.34 b	0.95 ± 0.01 ab	6.52 ± 0.40 b
T6	N_2_P_2_K_2_	1380.80 ± 302.08 ab	0.95 ± 0.04 ab	7.16 ± 1.65 ab
T7	N_2_P_3_K_2_	1281.24 ± 211.79 b	0.94 ± 0.02 ab	6.63 ± 0.71 b
T8	N_2_P_2_K_0_	1250.01 ± 214.96 b	0.96 ± 0.03 ab	7.09 ± 1.21 ab
T9	N_2_P_2_K_1_	1112.93 ± 7.42 b	0.94 ± 0.01 ab	6.69 ± 0.22 b
T10	N_2_P_2_K_3_	1042.75 ± 196.40 b	0.93 ± 0.02 b	6.24 ± 0.88 b
T11	N_3_P_2_K_2_	1150.40 ± 144.02 b	0.95 ± 0.01 ab	6.55 ± 0.60 b
T12	N_1_P_1_K_2_	1370.78 ± 336.40 ab	0.96 ± 0.04 ab	7.28 ± 1.69 ab
T13	N_1_P_2_K_1_	1171.17 ± 75.22 b	0.95 ± 0.01 ab	6.72 ± 0.47 b
T14	N_2_P_1_K_1_	1056.21 ± 77.82 b	0.96 ± 0.01 ab	7.02 ± 0.50 ab
R_N_	–	427.22	0.02	1.05
R_P_	–	332.58	0.01	0.64
R_K_	–	338.06	0.03	0.92
R_NP_	–	529.39	0.02	1.08
R_NK_	–	534.87	0.03	1.37
R_PK_	–	338.06	0.03	0.92
R_NPK_	–	887.22	0.05	2.13

Means ± SD with different letters indicate a significant difference among the 14 treatments, as determined by Duncan’s multiple range test (*p* < 0.05). R means the difference between the maximum and minimum values at different levels for single-factor, two-factor, and three-factor effects of N, P, and K fertilizer. R_N_ is the difference at 4 N levels (T2, T3, T6, and T11), R_P_ at 4 P levels (T4, T5, T6, and T7), R_K_ at 4 K levels (T8, T9, T6, and T10), R_NP_ at 8 NP levels (T2, T3, T4, T5, T6, T7, T11, and T12), R_NK_ at 8 NK levels (T2, T3, T6, T8, T9, T10, T11, and T13), R_PK_ at 8 PK levels (T4, T5, T6, T7, T8, T9, T10, and T14), and R_NPK_ at 14 NPK levels (T1–T14).

**Table 3:** Topological parameters of bacterial network of different NPK fertilization conditions.

Topo	NPK	NP	NK	PK	N	P	K
Number of Nodes	617	617	617	612	615	545	594
Number of Edges	3593	3593	3593	3444	3591	2447	3223
Closeness Centrality	0.61	0.61	0.61	0.60	0.61	0.56	0.60
Average Path Length	4.09	4.09	4.09	4.12	4.07	4.24	4.21
Transitivity	0.61	0.61	0.61	0.62	0.60	0.70	0.64
Modularity	0.75	0.75	0.75	0.76	0.75	0.73	0.76

Number of nodes: the number of nodes in the target network; number of edges: the number of edges in the target network; closeness centrality: the ratio of the average distance between the target node and all other nodes; average path length: the sum of all the short paths in the network; transitivity: the probability of connecting the target node and the adjacent node; modularity: the modularity index.

**Table 4:** The correlation analysis of dominant phylum bacteria and dominant genera bacteria.

Species	Actinobacteria	Chloroflexi	Acidobacteria	Proteobacteria
*Cupriavidus*	0.024	0.105	0.063	−0.038
*Methylobacterium*	−0.703 **	−0.659 **	−0.689 **	0.855 **
*Brevundimonas*	−0.659 **	−0.495 **	−0.496 **	0.651 **
*Sphingobium*	−0.626 **	−0.350 *	−0.500 **	0.643 **
*Acinetobacter*	−0.641 **	−0.442 **	−0.617 **	0.732 **
*Sphingomonas*	−0.157	−0.132	−0.370	0.298

*, ** indicate significant (*p* < 0.05) and highly significant (*p* < 0.01), respectively.

**Table 5:** Correlation analysis between key bacteria and soil chemical characteristics.

Species	pH	N Content	P Content	K Content
*Methylobacterium*	−0.383 *	0.212	0.141	0.140
*Brevundimonas*	−0.219	0.322 *	0.178	0.080
*Sphingobium*	−0.154	0.240	0.165	0.293 *
*Acinetobacter*	−0.356 *	0.433 **	0.403 **	0.080

*, ** indicate significant (*p* < 0.05) and highly significant (*p* < 0.01), respectively.

**Table 6:** The “3414” fertilization experiment rates [48].

No.	Treatment ^1^	N ^1^ (g·Plant^−1^)	P_2_O_5_ ^1^ (g·Plant^−1^)	K_2_O ^1^ (g·Plant^−1^)
T1	N_0_P_0_K_0_	0 (0)	0 (0)	0 (0)
T2	N_0_P_2_K_2_	0 (0)	2 (0.1332)	2 (0.356)
T3	N_1_P_2_K_2_	1 (0.266)	2 (0.1332)	2 (0.356)
T4	N_2_P_0_K_2_	2 (0.532)	0 (0)	2 (0.356)
T5	N_2_P_1_K_2_	2 (0.532)	1 (0.0666)	2 (0.356)
T6	N_2_P_2_K_2_	2 (0.532)	2 (0.1332)	2 (0.356)
T7	N_2_P_3_K_2_	2 (0.532)	3 (0.1998)	2 (0.356)
T8	N_2_P_2_K_0_	2 (0.532)	2 (0.1332)	0 (0)
T9	N_2_P_2_K_1_	2 (0.532)	2 (0.1332)	1 (0.178)
T10	N_2_P_2_K_3_	2 (0.532)	2 (0.1332)	3 (0.534)
T11	N_3_P_2_K_2_	3 (0.798)	2 (0.1332)	2 (0.356)
T12	N_1_P_1_K_2_	1 (0.266)	1 (0.0666)	2 (0.356)
T13	N_1_P_2_K_1_	1 (0.266)	2 (0.1332)	1 (0.178)
T14	N_2_P_1_K_1_	2 (0.532)	1 (0.0666)	1 (0.178)

^1^ Treatment numbers represent no (0), low (1), medium (2), and high (3) levels of fertilization, respectively (values in parentheses represent a specific amount of fertilization). Data from Yang et al., 2020 [48].

**Table 7:** Biomass and macro-element accumulations of *Phoebe bournei* seedlings under different NPK fertilization conditions.

Number	Treatment	Plant Biomass (g·Plant^−1^) [48]	N Accumulation (mg·Plant^−1^)	P Accumulation (mg·Plant^−1^)	K Accumulation (mg·Plant^−1^)
T1	N_0_P_0_K_0_	2.89 ± 0.23 e	31.43 ± 2.05 g	9.14 ± 0.56 g	78.36 ± 10.72 i
T2	N_0_P_2_K_2_	2.73 ± 0.27 e	39.87 ± 4.31 f	11.80 ± 1.39 ef	132.07 ± 11.24 fg
T3	N_1_P_2_K_2_	4.01 ± 0.32 c	55.50 ± 4.24 d	14.98 ± 0.32 d	158.46 ± 3.14 de
T4	N_2_P_0_K_2_	4.97 ± 0.14 b	62.71 ± 1.22 bc	12.72 ± 0.22 e	115.07 ± 1.71 h
T5	N_2_P_1_K_2_	4.10 ± 0.07 c	64.96 ± 0.84 b	16.27 ± 3.25 cd	220.64 ± 10.78 b
T6	N_2_P_2_K_2_	7.27 ± 0.41 a	82.45 ± 4.85 a	24.68 ± 0.97 a	323.91 ± 16.26 a
T7	N_2_P_3_K_2_	4.71 ± 0.34 b	58.85 ± 4.84 cd	15.69 ± 0.75 cd	120.47 ± 5.48 gh
T8	N_2_P_2_K_0_	3.95 ± 0.14 c	54.39 ± 0.70 d	11.08 ± 0.38 ef	91.79 ± 11.29 i
T9	N_2_P_2_K_1_	3.97 ± 0.20 c	64.83 ± 2.05 bc	16.09 ± 0.18 cd	145.80 ± 0.75 ef
T10	N_2_P_2_K_3_	3.30 ± 0.12 d	44.73 ± 5.19 ef	10.40 ± 0.38 fg	167.34 ± 7.97 cd
T11	N_3_P_2_K_2_	2.73 ± 0.16 e	47.73 ± 3.81 e	12.79 ± 0.79 e	111.10 ± 6.32 h
T12	N_1_P_1_K_2_	4.65 ± 0.32 b	62.46 ± 1.05 bc	17.07 ± 0.48 bc	180.19 ± 2.11 c
T13	N_1_P_2_K_1_	4.69 ± 0.12 b	65.76 ± 3.05 b	18.78 ± 0.99 b	175.76 ± 7.18 c
T14	N_2_P_1_K_1_	4.20 ± 0.12 c	64.04 ± 1.24 bc	12.52 ± 1.13 e	133.77 ± 7.29 fg

Means ± SD with different letters indicate a significant difference among the 14 treatments, as determined by Duncan’s multiple range test (*p* < 0.05). Data from Yang et al., 2020 [48].

## Data Availability

The datasets used and analysed during the current study could be available from the corresponding author on reasonable request.
